# Hemodynamics and Drinking in the Giraffe

**DOI:** 10.1111/apha.70046

**Published:** 2025-04-22

**Authors:** Christian Aalkjær, Mads Damkjær, Ulrik T. Baandrup, Mads F. Bertelsen, Torbjørn Brøgger, Emil Brøndum, Carl C. Danielsen, Jonas A. Funder, Carsten Grøndahl, J. Michael Hasenkam, Per G. Henriksen, Niels H. Secher, Nini Skovgaard, Morten H. Smerup, Niklas Telinius, Kristine H. Østergaard, Peter Bie, Tobias Wang

**Affiliations:** ^1^ Department of Biomedicine and Danish Cardiovascular Academy Aarhus University Aarhus Denmark; ^2^ Department of Molecular Medicine University of Southern Denmark Odense Denmark; ^3^ Department of Paediatrics and Adolescent Medicine, Lillebaelt Hospital University Hospital of Southern Denmark Kolding Denmark; ^4^ Department of Pathology Aalborg University Hospital Aalborg Denmark; ^5^ Copenhagen Zoo Frederiksberg Denmark; ^6^ Department of Clinical Medicine Aarhus University Aarhus Denmark; ^7^ Department of Surgery, Faculty of Health Sciences University of the Witwatersrand Johannesburg South Africa; ^8^ Section for Zoophysiology, Department of Biology Aarhus University Aarhus Denmark; ^9^ Department of Anesthesia, Rigshospitalet, Department of Clinical Medicine University of Copenhagen Copenhagen Denmark; ^10^ SOSU FVH Vejle Denmark; ^11^ Faculty of Health Sciences University of Copenhagen Copenhagen Denmark; ^12^ Department of Biology Aarhus University Aarhus Denmark

**Keywords:** blood pressure, giraffes, gravitational physiology, hypertension

## Abstract

**Background:**

The circulation of 4–6 m tall giraffes is markedly affected by gravity. To ensure cerebral perfusion, upright giraffes generate a blood pressure in excess of 200 mmHg. Before drinking, the head is lowered by 3–5 m, providing exceptional hemodynamic challenges. Here, we provide quantitative hemodynamic measures during head movement and drinking.

**Methods:**

We measured carotid pressure, jugular pressure, heart rate, and blood flow in awake giraffes, along with circulating blood volume and cerebrospinal fluid pressure in anesthetized giraffes. We also analyzed the contractility and innervation of isolated cerebral and extracranial arteries, and the mechanical properties of jugular veins.

**Results:**

When heads were lowered for drinking (i) blood pressure at heart level decreased but increased again during drinking, (ii) jugular pressure increased and oscillated during drinking, (iii) heart rate fell, (iv) carotid blood flow was unchanged, while cephalic hemodynamic resistance increased, and (vi) cranial cerebrospinal fluid pressure increased. Small cerebral arteries exhibited strong myogenic responses, particularly at around 100 mmHg, while extracranial arteries responded at higher pressures (200–250 mmHg). The giraffe's blood volume was small and blood pressure sensitive to minor reductions in blood volume.

**Conclusions:**

Central blood pressure decreased when the head was lowered, but drinking per se caused a surprising rise in blood pressure to pre‐drinking levels. This rise in blood pressure is likely due to the transfer of esophageal water boli acting on the jugular veins. The cephalic capillaries are protected by a strong myogenic response and sympathetic innervation.

## 1 Introduction

Gravitational force affects arterial and venous pressures of all terrestrial vertebrates, and these effects are obviously exacerbated in tall animals. The hemodynamic disturbances imposed by changes in body position are alleviated by a plethora of physiological countermeasures, including changes in vascular resistances and cardiac performance mediated by neuroendocrine mechanisms. In species with natural head positions above heart level, the heart must supply the brain with blood against an orthostatic pressure gradient, which is proportional to the heart‐to‐brain vertical distance. This is particularly challenging in adult giraffes, where this distance can be 1.5–3 m. This explains why blood pressure at heart level in giraffes is twice that of other mammals. However, while providing cerebral perfusion pressures similar to those of other mammals, this high aortic pressure represents a severe hemodynamic challenge when a giraffe lowers its head to drink [1, 2, 3, 4, 5, 6]. The rapid lowering of the head to the ground is expected to triple cephalic arterial pressure, and a pressure above 300 mmHg has been measured in the distal carotid artery of a single drinking giraffe [5]. It remains virtually unknown how the circulation of the giraffe adjusts to such abrupt elevations of cerebral arterial pressures and how vascular catastrophes are avoided. Moreover, it is not known whether cerebral perfusion is maintained when giraffes drink and when they subsequently, within a second, raise their heads 4–5 m above the ground.

Here, we capitalize on an array of experimental approaches to understand how pressure and flow in the neck and head of giraffes are regulated. We asked whether giraffes rely on well‐known mechanisms when they lower their heads to drink or whether the species has evolved novel solutions. In connection with drinking in a natural setting, implanted data‐loggers measured pressures in the proximal and distal ends of the carotid artery and jugular vein together with carotid blood flow. Blood accumulates in the jugular veins when the head is lowered in anesthetized giraffes [7] and this redistribution of blood seems to reduce cardiac preload and pressure generation via the Frank–Starling mechanism [7, 8]. Here, we therefore assessed whether this also occurs in awake giraffes in a natural setting, and we assess how blood volume and vascular compliance contribute to the sensitivity of blood pressure when blood is sequestered in the jugular veins during drinking. We further investigated the myogenic and pharmacological responses of isolated cephalic small arteries to understand how hydrodynamic resistance is controlled in the cephalic circulation during the lowering of the head. Finally, the pressure in the cerebrospinal fluid was measured when the head was lowered in anesthetized giraffes to quantify the extent to which increases in this pressure counterbalance the rise in arterial pressure within the skull.

Overall, we demonstrate that several independent mechanisms act synergistically to maintain adequate cardiovascular function when giraffes perform the largest vertical head displacement of any animal on Earth.

## 2 Materials and Methods

### 2.1 Studies in Freely Moving Giraffes

#### 2.1.1 Animals and Ethical Permits

Data were obtained from 13 healthy male southern giraffes ( *Giraffa camelopardalis giraffa* ) which were acclimatized to the experimental environment for 3–8 weeks at a quarantine facility in Hammanskraal, South Africa. Experimental procedures were approved by the Animal Ethics Screening Committee at the University of Witwatersrand, Johannesburg, and the Animal Use and Care Committee at the University of Pretoria, Pretoria, South Africa. Permission to euthanize the animals was granted by the Gauteng Province, South Africa, and the studies were overseen by the Animal Ethics Committee/Animal Use and Care Committee, University of Pretoria. An additional six healthy giraffes (hybrids, four males) from a Danish zoo were instrumented prior to culling carried out for population management purposes [9]. The experiments were approved by the Animal Experimentation Council, Ministry of Food, Agriculture and Fisheries of Denmark. The 19 giraffes were 42 ± 38 months old, 3.66 ± 0.28 m tall, and weighed 505 ± 102 kg (mean ± SD).

#### 2.1.2 Anesthesia and Hemodynamics

Giraffes were deprived of food and water overnight and premedicated with medetomidine (6 μg/kg) by remote injection (DanInject, Kolding, Denmark). After about 15 min, the sedated giraffes were provided with a halter and blindfolded, and anesthesia was induced with etorphine (9 μg/kg) and ketamine (0.9 mg/kg) i.m. A rope connected to the halter allowed control of the head when the giraffes became recumbent. Upon induction, the trachea was intubated orally using a cuff (ID 20 mm) and assisted ventilation was provided with 100% oxygen using a Hudson demand valve (Hudson RCI, Teleflex Medical, Morrisville, NC, USA). The animals were placed in right lateral recumbency with the head elevated approximately 1 m above heart level. Electrocardiogram, rectal temperature, end‐tidal carbon dioxide tension, arterial blood pressure, and arterial oxygen saturation were monitored (Mindray PM9000Vet, E‐Vet, Haderslev, Denmark). Arterial and venous blood gas variables were monitored every 10 min using a GEM Premier 3500 analyzer (Instrumentation Laboratory, Bedford, MA, USA). Local analgesia (2% lidocaine and 0.5% bupivacaine) was applied to the site of skin incision and ketoprofen (3 mg/kg) administered i.v. Anesthesia was maintained by etorphine (0.45 μg/kg/h) and ketamine (0.5 mg/kg/h) until completion of surgery. One liter Macrodex 60, 2 L lactated Ringer's solution, and 0.5 L dextrose 50% were provided i.v. to stabilize the circulation.

#### 2.1.3 Pressure, Flow, and Body Movement

During general anesthesia, a datalogger with intravascular pressure catheters, a perivascular flow probe, a memory card, and an accelerometer (all from EndoSomatic Systems, Davis, CA, USA) were implanted. The pressure catheters were inserted proximally (40–50 cm above the base of the heart) and distally (10–15 cm below the base of the skull) in the carotid artery and left external jugular vein (hereafter “jugular vein”) through incisions on the left side of the base of the neck. After heparinization (150 IU/kg), the artery and the vein were clamped for insertion of catheters at the proximal position. In four giraffes, additional catheters were positioned distally in the carotid artery and jugular vein (distal) using a custom‐built tunneling rod and two additional incisions along the neck. The distal and proximal catheters were 1–1.5 m apart. The distal catheters were connected to an implant in a subcutaneous pocket at the proximal incision site. Two‐point calibration, 0–200 mmHg (arterial) and 0–50 mmHg (venous) was performed before implantation. Mean pressures were calculated by integration of the pressure recordings over 2–5 cycles. The precalibrated perivascular flow probe was placed at the distal position. The accelerometer was placed at the distal position. Anesthesia was reversed (Atipamezole 10 mg i.m. and 10 mg i.v., Naltrexone 100 mg i.v., Doxapram 400 mg i.v.) and the giraffes supported in sternal recumbency until ready to stand.

After overnight recovery from surgery, the giraffes were released in natural settings for continuous measurements over the next 3–10 days during which they were able to move, forage, and drink of their own volition.

#### 2.1.4 Video Surveillance

In South Africa, the giraffes were kept in a 100 m × 100 m enclosure, including a pond, and observed via two video cameras (KCM‐7211 m ACTi, Taipei, Taiwan). In Denmark, the giraffes were kept within a 50 m x 50 m enclosure adjacent to a 145 m^2^ stable. Two video cameras (TV‐IP310PI, TRENDnet, Torrance, CA) covered an indoor water trough. Video sequences of drinking and head movements from upright to a near horizontal position were identified and (for observations in South Africa) aligned with accelerometer data. The following variables were measured or derived over 2–10 heart cycles: arterial pressure (systolic, diastolic, and mean), venous pressure (mean and peak), heart rate, carotid artery blood velocity, and an index of vascular resistance, that is, arterial minus venous mean pressure divided by flow velocity. As we have shown previously, the carotid artery diameter is unaffected by changes in the head position [7], therefore, flow velocity changes provide a relevant measure of volume flow changes, which is needed for calculation of vascular resistance.

Memory card data were transferred to a computer using Rich Text Format editor (Microsoft RTF v1.8) to preserve formatting and time stamps. Data were then converted from binary to ascii format using a custom developed data‐logger file converter and subsequently imported to LabChart 7 (ADI Instruments, Bella Vista, NSW, Australia). The analog data were calibrated to actual pressure and flow values using the two‐point calibrations already recorded on the memory data cards.

### 2.2 Studies in Anesthetized Giraffes

#### 2.2.1 Anesthesia and Suspension

The giraffes were anesthetized with the medication described above, and the body position was controlled as described by Brøndum et al. [7]. Anesthesia was maintained by α‐chloralose i.v. at 30 mg/kg initially and approximately 3 mg/kg/h depending on reflexes and breathing pattern. Vascular sheaths were placed as described. Straps were placed around each limb, leaving the thoracic and abdominal regions free of external pressure, and the giraffe was hoisted to an upright position (see photo in figure 1 of Brøndum et al. [7]).

#### 2.2.2 Pressure Recordings

Blood pressure was measured by catheter‐tip transducers (5 Fr Micro‐Tip SPC 350, Millar Instruments, Houston, TX, USA) advanced to the aortic arch for mean arterial pressure (MAP). Cerebrospinal fluid pressure was measured in three giraffes by puncture of the cisterna magna. Pressures were sampled at 100 Hz (Biopack MP100 data acquisition system; AqKnowledge 3.7.2; Biopack Systems, Goleta, CA, USA).

#### 2.2.3 Blood Volume Determination

Plasma volume was determined by dilution of Evans Blue (EB) by use of on‐site analyses, individual standard curves, log‐linear intercept determination of initial concentration, and correction for trapped plasma (for details, see Text S1).

#### 2.2.4 Hypovolemia

After stabilization of cardiovascular variables and determination of blood volume, blood was withdrawn in aliquots of 500 mL from the jugular vein catheter until 10% of the calculated BV had been removed. During bleeding, central hemodynamics were recorded as described above.

### 2.3 In Vitro Studies of Blood Vessels

#### 2.3.1 Vascular Compliances and Composition

The load‐strain relationship of jugular veins was determined in specimens obtained from seven giraffes as described [10] allowing calculation of vascular compliances (relative volume change per unit change in applied transmural pressure, see Text S1). In addition, vascular elastin and collagen contents were determined (see Text S1).

#### 2.3.2 Function of Small Cephalic Arteries

Following euthanasia, biopsies from brain parenchyma near the middle cerebral artery, the tongue, and a muscle in the upper neck were obtained. Small arteries were dissected under a stereomicroscope and mounted in a myograph placed on an inverted microscope for investigation of myogenic and agonist‐induced changes in vascular tone (for details, see Text S1).

#### 2.3.3 Innervation of Cerebral Blood Vessels

Small cerebral arteries, one from each of five giraffes, were dissected and prepared for microscopy. After neutral buffered formalin fixation, the arteries were aligned in agar ditches for cross‐sectional slicing. Following paraffin embedding, two‐micron thick sections were cut and stained by hematoxylin–eosin (HE) and elastic van Gieson reaction. Sympathetic innervation was studied immunohistochemically using anti‐tyrosine hydroxylase antibody (ab112, Abcam, Cambridge, UK).

### 2.4 Statistics

Values are presented as mean ± SEM unless otherwise indicated, “n” denotes number of animals. The hemodynamic effect of changing head position was assessed by comparison to the steady state by a paired *t*‐test. Data including multiple mean values were analyzed by ANOVA adjusted for repeated measures when appropriate; significant differences were identified by Tukey's test. *p* < 0.05 was taken to represent a significant change.

## 3 Results

**FIGURE 1:**
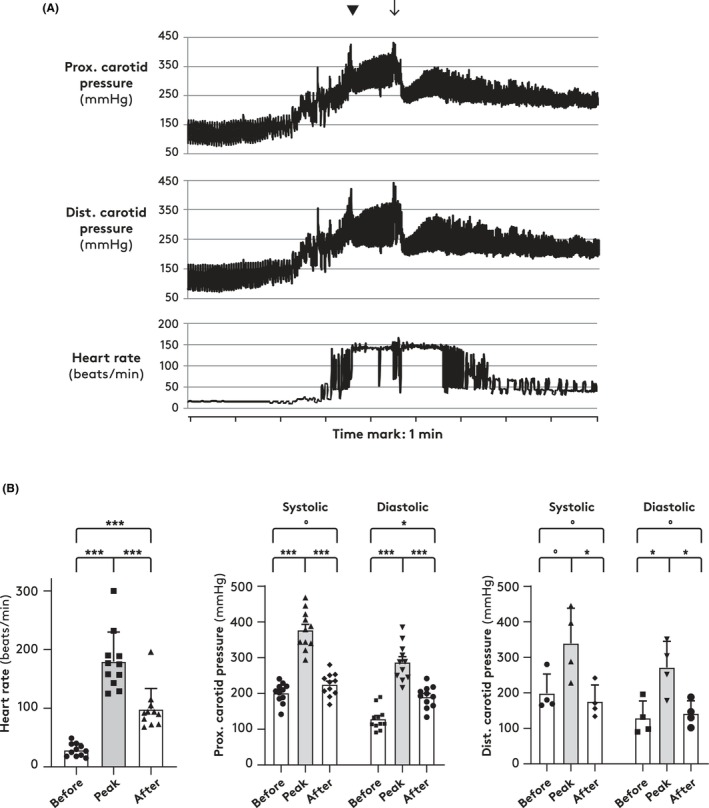
(A) Proximal and distal carotid pressures and heart rate in a giraffe rising from sternal to upright position after anesthesia. Triangle indicates failed attempt to rise; arrow indicates successful attempt to rise. (B) Mean and SEM values for heart rate (left panel, *n* = 11) and for systolic and diastolic carotid pressures at proximal (middle panel, *n* = 11), and distal (right panel, *n* = 4) catheters before and after standing up and at the peak hemodynamic changes. ANOVA for repeated measures followed by Tukey's test; *p < 0.05, ***p < 0.005, ^o^ *p* > 0.05.

Postoperatively, arterial pressure and heart rate varied considerably with posture. After reversal of anesthesia, giraffes were placed in a sternal position at which blood pressures and heart rates were relatively stable (example, Figure 1A). Subsequently, resumption of normal standing position included increases in carotid pressure (exceeding 400 mmHg in some individuals) and heart rate (from 30–40 to 100–200 bpm) (Figure 1B).

### 3.1 Freely Moving Giraffes: Hemodynamic Changes During Head Movement

**FIGURE 2:**
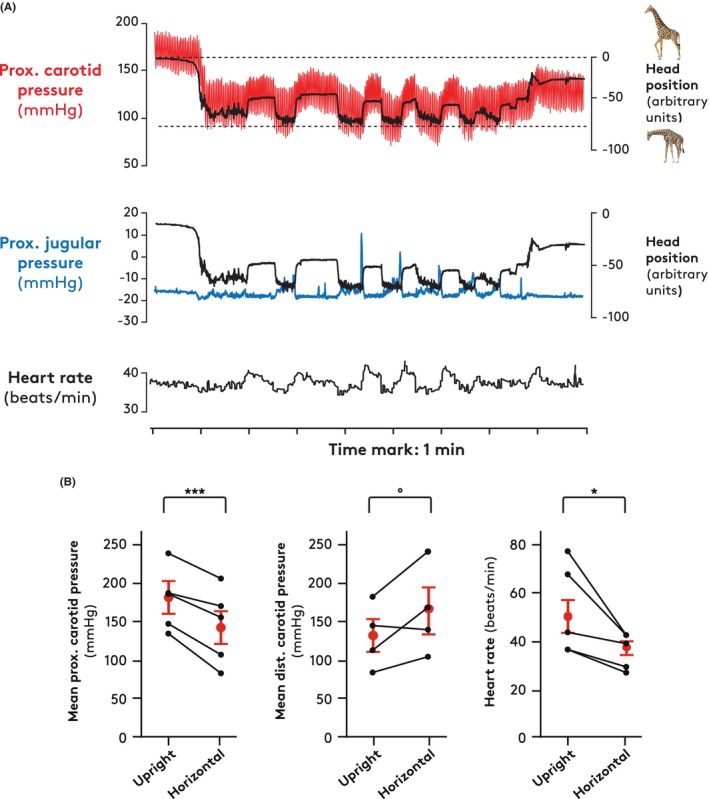
(A) Proximal carotid pressure (red), proximal jugular pressure (blue) and heart rate of an instrumented giraffe moving its head repeatedly from the upright position to a near horizontal position. Head position (black) shown on right axis; at the lowest position values the neck is horizontal indicated by the lower hatched line; at the highest values the neck is in an upright position indicated by the upper hatched line. The tracing is typical for recordings obtained in five giraffes. (B) Mean proximal and distal carotid pressures and heart rate in upright and horizontal position in four giraffes. Mean values and SEM in red. Paired *t*‐test of pressure and heart rate changes: *p < 0.05, ***p < 0.005, ^o^ *p* > 0.05.

Under natural conditions, the giraffe often moves the neck from upright (about 80°) to near horizontal positions. In our giraffes, this behavior was associated with decreases in proximal carotid pressure (example, see Figure S1) and heart rate, which in five giraffes averaged 33 ± 5 mmHg and 14 ± 5 beats/min, respectively (Figure 2A,B). In two giraffes, in which heart rates in the upright position were near 70 beats per min, the bradycardia was pronounced. During leveling of the neck, distal carotid pressures increased in three of four individuals (Figure 2B, middle panel). Proximal jugular pressures did not change systematically. Subsequently, when the head was lifted, a sharp, transient increase in jugular venous pressure was sometimes seen (examples in Figure 2A).

### 3.2 Freely Moving Giraffes: Hemodynamic Changes Associated With Drinking

#### 3.2.1 Proximal Carotid Artery

**FIGURE 3:**
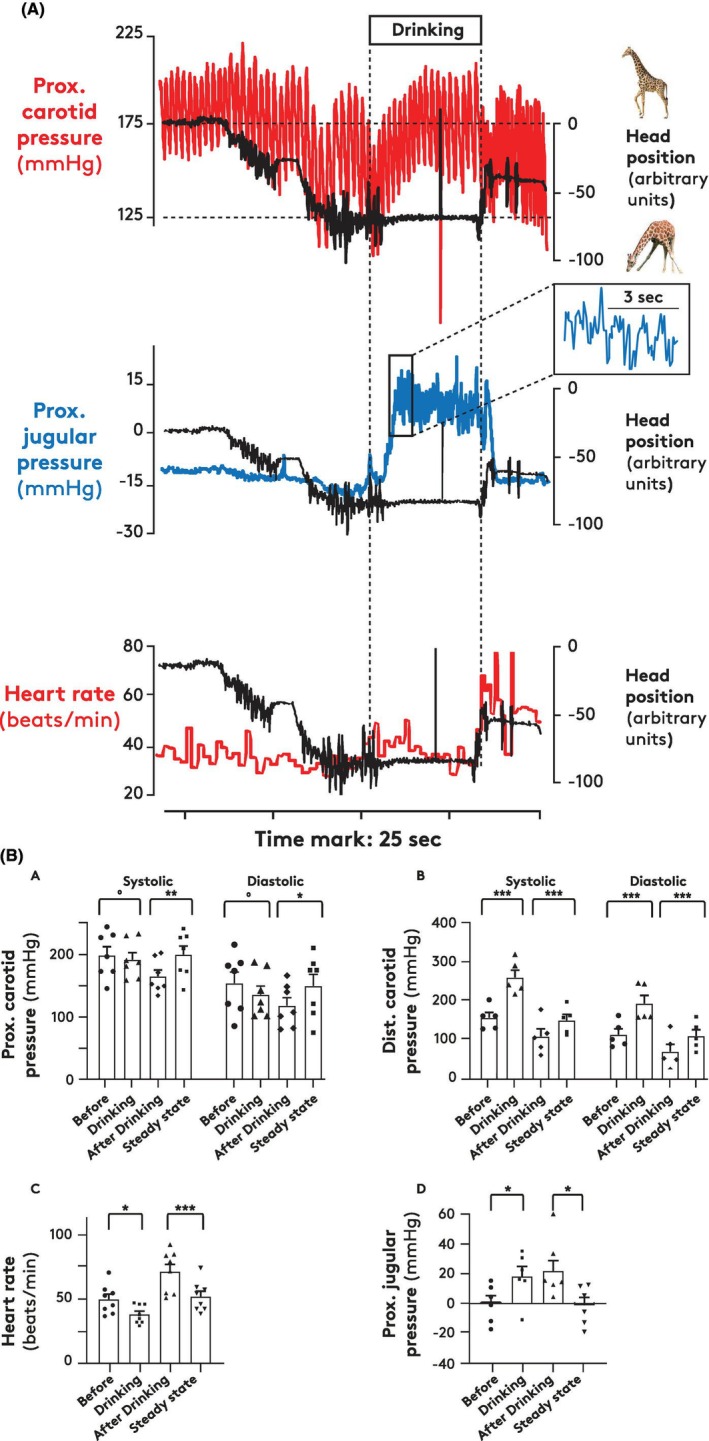
(A) *Upper panel*: Changes in proximal carotid pressure (red line, left axis) occurring in relation to changes in head position (black line, right axis). *Middle panel*: Changes in proximal jugular venous pressure (blue line, left axis) occurring in relation to changes in head position (black line, right axis). *Lower panel*: Changes in heart rate (red line, left axis) occurring in relation to changes in head position (black line, right axis). For “Head position”, maximal and minimal values correspond to erect and ground level positions, respectively (indicated by hatched lines in upper panel). The insert shows the jugular venous pressure at an expanded timescale. Tracings are unedited; sudden, large excursions shortly after middle of drinking period in all three panels represent artifacts. (B) Mean and SEM of proximal carotid systolic and diastolic pressures (A), distal carotid systolic and diastolic pressures (B), heart rate (C), and proximal jugular pressures (D) at steady state before drinking in upright position (“Before”), at the end of a drinking head down position (“Drinking”), at the peak hemodynamic transient associated with lifting of the head after drinking (“After drinking”), and at steady state after drinking in upright position (“Steady state”). Each data point represents the mean value of multiple (average: 9) drinking episodes in each giraffe. Difference between means was tested with paired *t*‐test; *p < 0.05, **p < 0.01, ***p < 0.005, ^o^ *p* > 0.05.

When the head was lowered for drinking, mean proximal carotid pressure initially decreased by 24 ± 6 mmHg (*n* = 7 giraffes, Figure S2; examples in Figures 3A and S3) much like the change occurring when the neck was moved to a near‐horizontal position (Figures 2A and S1). However, during drinking—that is, with the head at hoof level—these decreases subsided in most giraffes (example Figure 3A), such that mean proximal carotid pressure approached the preceding upright values (Figure S2). When the head was lifted after drinking, proximal carotid pressures fell initially but returned to upright steady state values within 10–20 s (Figure 3B). In one giraffe, we serendipitously measured proximal carotid pressure while the animal lowered the head to drink from an empty water reservoir; in this case, the marked decline in proximal carotid pressure (~50 mmHg) persisted throughout the period of apparent intention to drink (Figure S3).

#### 3.2.2 Distal Carotid Artery

As expected, distal carotid systolic and diastolic pressures increased when the head was lowered to drink and remained elevated during drinking (Figure 3B, example Figure S4). When the head was raised after drinking, there was a transient (5–10 s) decrease in distal carotid pressure to values below the steady state pressure before steady state pressure was regained over 10–20 s (Figures 3B and S4).

#### 3.2.3 Jugular Vein

During drinking, the pressure in the proximal jugular vein increased; in seven giraffes by 20 ± 6 mmHg (Figure 3B, see also Figures 3A and S4) showing clear oscillations in six of the seven giraffes (Figures 3A and S4). In one giraffe, distal jugular pressure was also measured (Figure S4); this was markedly higher than the corresponding proximal jugular pressure, and during drinking, it increased by some 70 mmHg, oscillating much like the corresponding proximal jugular pressure. The frequencies of these oscillations were higher than the heart rate in 21 of 24 observations and lower in the other three (Figure S5). The amplitude was variable (1–20 mmHg). Pressure variation in jugular veins synchronous with heart rate was not observed. When the head was raised after drinking, a small transient increase in pressure was sometimes seen, but it was less conspicuous than the transient in jugular pressure seen when the head was lifted from the horizontal position (Figures 2A and 3A).

#### 3.2.4 Carotid Flow and Vascular Resistance

**FIGURE 4:**
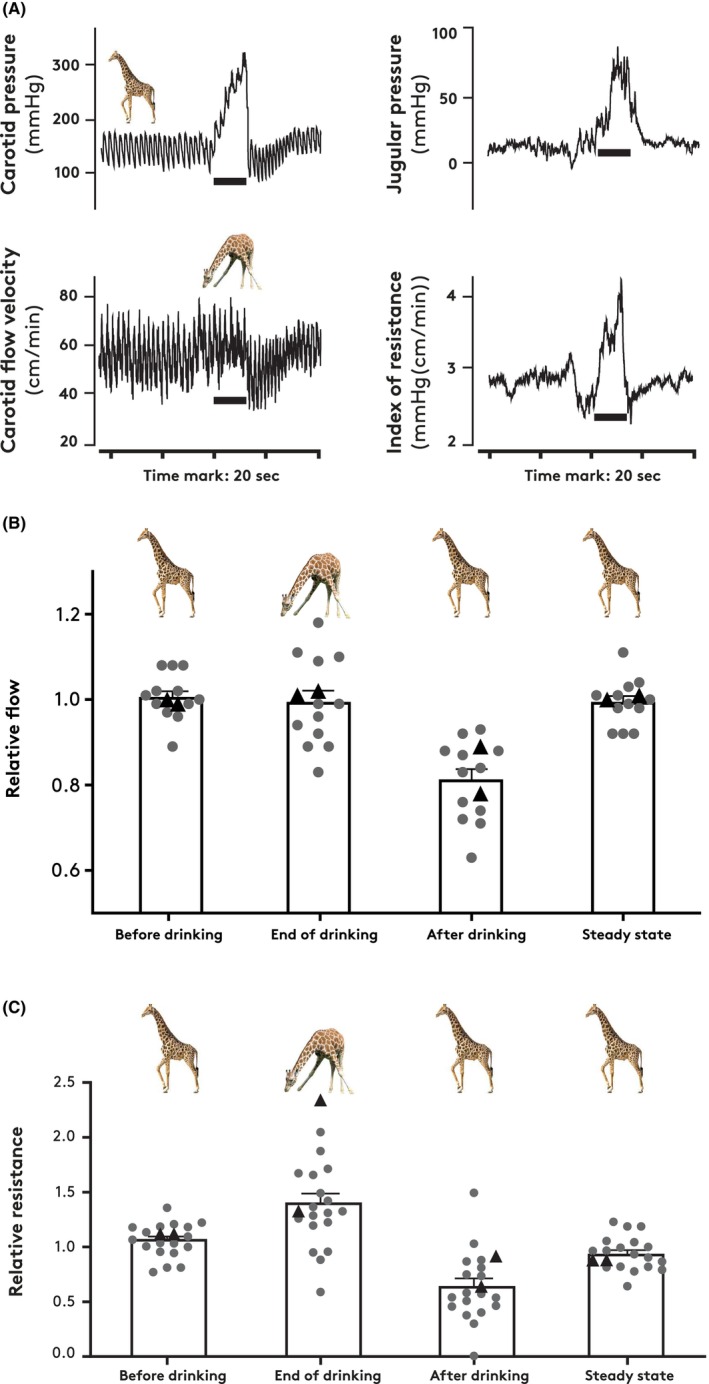
(A) Carotid and jugular pressures and carotid flow halfway up the neck in connection with an episode of drinking (one giraffe). Horizontal black bars indicate active drinking. Vascular resistance is calculated from carotid pressure and flow data. (B and C) Vascular flow velocity and vascular resistance, respectively, from 12 drinking episodes in one giraffe (dots) and two drinking episodes in another giraffe (triangles). Data are normalized to the mean value of “Before” drinking and “Steady state” after drinking.

In two giraffes, we measured flow velocity in the carotid artery (Figure 4A). Flow velocity was unaffected when the giraffes were drinking and transiently reduced (ca. 20% at peak reduction) over a 10–15 s period when the head was lifted (Figure 4A,B). With concomitant pressure data, these results enabled the assessment of relative changes in cephalic vascular resistance (Figure 4A). In both giraffes, the index of resistance increased by the end of drinking (Figure 4C) and, upon lifting of the head, decreased immediately to a level lower than the steady state value measured before drinking (Figure 4C).

#### 3.2.5 Heart Rate

At the end of drinking, prior to head elevation, heart rate was reduced (−12 ± 4 bpm, Figure 3B, graph C) compared to upright control (≈50 bpm). When the head was raised, tachycardia appeared within a few seconds; subsequently, heart rate returned (−21 ± 4 beats/min within 10–30 s) to the upright control value (Figure 3B, graph C).

### 3.3 Anesthetized Giraffe: Cerebrospinal Fluid Pressure During Head Movement

In three giraffes, cerebrospinal fluid pressures (cisterna magna) were measured while the neck was passively moved from a near‐horizontal position to an upright position. This was associated with a linear decrease in cerebrospinal fluid pressure, which did not follow the pressure changes expected due to gravity in an open system (Figure S6).

### 3.4 Anesthetized Giraffes: Blood Volume and the Effect of Volume Reduction

**FIGURE 5:**
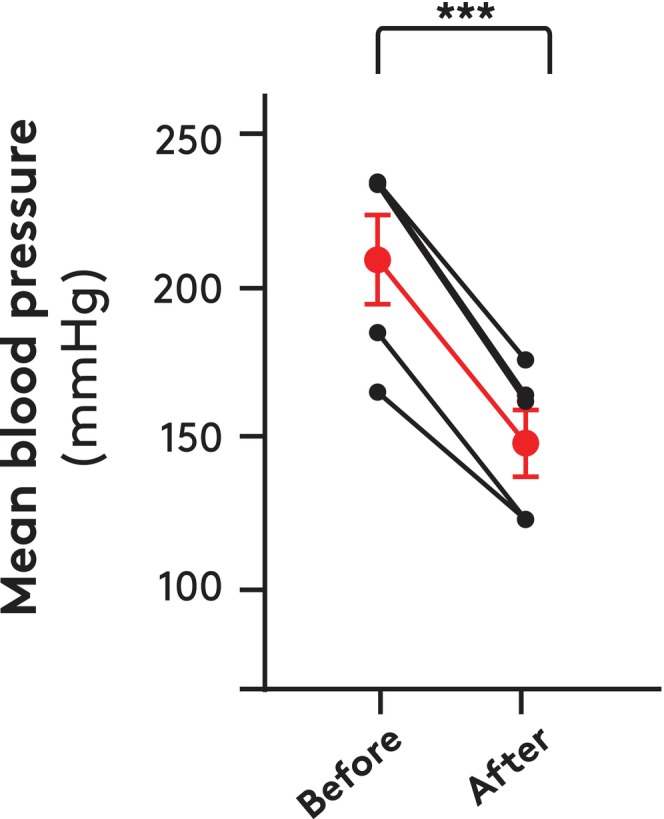
Effect on mean arterial blood pressure of withdrawal of 10% of measured total blood volume from five anesthetized giraffes. Mean values and SEM are indicated in red. Paired *t*‐test; ***p < 0.005.

In 12 giraffes (body mass 511 ± 31 kg, height 3.6 ± 0.1 m), hematocrit, plasma osmolality, and sodium and potassium concentrations measured 43% ± 2%, 330 ± 4 mOsm/kg, 138.0 ± 1.3, and 4.1 ± 0.2 mmol/L, respectively. Plasma and blood volumes were 34 ± 2 and 56 ± 3 mL/kg body mass, respectively. In five animals, blood volume was measured and thereafter reduced by ≈10% by bleeding over ≈10 min while the pressure in the proximal carotid artery was recorded. The mean proximal carotid pressure decreased by 30% (*p* < 0.001) when blood volume was reduced (Figure 5).

### 3.5 In Vitro: Physical Characteristics of Giraffe Jugular Veins

**FIGURE 6:**
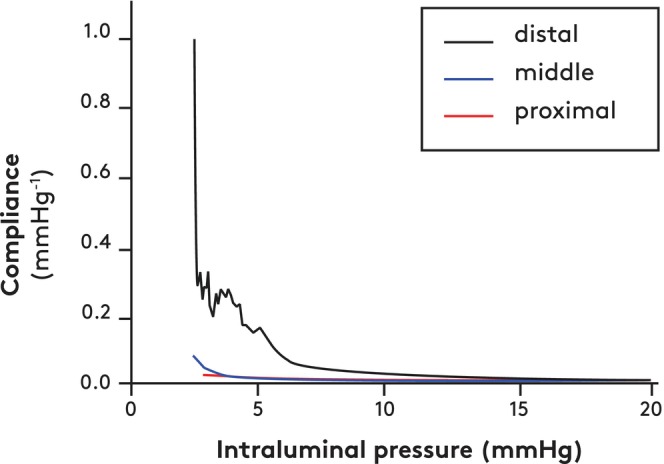
Calculated compliance of the jugular vein. Data based on load‐strain relationships and calculated intraluminal pressures (see Methods S1 and Figure S7). Load‐strain measurements were performed on three 2‐mm specimens from each of three locations, proximal, middle and distal, in seven giraffes.

The middle and distal segments of the giraffe jugular vein withstood higher loads before rupture than the proximal segment (Figure S7A, *p* < 0.05 and *p* < 0.001, respectively), and the distal segment distended more before rupture (maximal strain) than the proximal and middle segments (Figure S7A, *p* < 0.001). Remarkably, when intraluminal pressure increased from 3 to 10 mmHg, calculated volumes of the middle and proximal segments of the jugular veins increased less than 15% (Figure S7B) in contrast to much larger volume changes of similar pressure increases in the distal segments (Figure S7B). Consequently, the derived compliance values for the distal jugular vein segment were much higher than those calculated for the proximal and middle segments at low pressures (Figure 6). At intraluminal pressures higher than 10 mmHg, compliance was low for all segments.

Jugular venous elastin and collagen contents showed regional differences. From proximal to distal neck locations, the elastin content (% dry weight) increased significantly (Table 1). Thus, the elastin/collagen ratio increased markedly from proximal to distal locations and paralleled the increase in compliance along the jugular vein.

**TABLE 1:** Elastin and collagen contents in giraffe jugular veins (% dry weight).

Location	Elastin (%)	Collagen (%)	Elastin/collagen ratio
Proximal	6.4 ± 0.6	66.3 ± 2.2	0.097
Middle	8.1 ± 0.9	62.4 ± 3.3	0.130
Distal	10.6 ± 0.5	57.9 ± 3.0	0.183

### 3.6 In Vitro: Myogenic Responses and Responses to Agonists in Small Arteries From the Head

**FIGURE 7:**
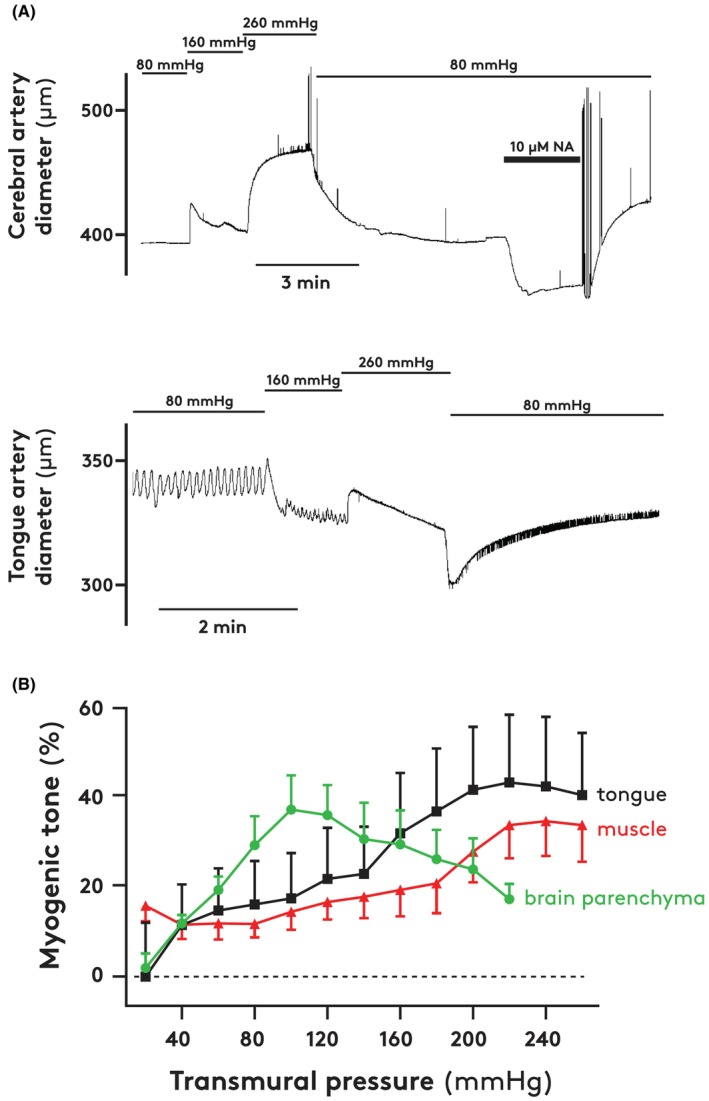
(A) Diameter of isolated pressurized cerebral (upper panel) and tongue (lower panel) arteries. The intraarterial pressures were changed as indicated and the cerebral artery was stimulated with 10 μM noradrenaline (NA) as indicated. (B) Myogenic tone as a function of intraarterial pressure in isolated small arteries from the brain parenchyma (*n* = 5), tongue (*n* = 6), and upper neck muscle (*n* = 8). Vertical bars indicate SEM.

In Ca^2+^‐free PSS at a transmural pressure of 80 mmHg, the diameters of the small cerebral, tongue, and muscle arteries were 394 ± 28 (*n* = 5), 346 ± 42 (*n* = 6), and 217 ± 31 μm (*n* = 8), respectively. The cerebral and tongue arteries responded to an increased intra‐arterial pressure with constriction (myogenic response; Figure 7A, upper and lower traces, respectively). Notably, the cerebral arteries did not constrict when pressure was increased from 160 to 260 mmHg; the highest myogenic tone was found at a transmural pressure of about 100 mmHg, compared to about 250 mmHg for the other arteries (Figure 7A,B). All arteries exhibited pronounced myogenic tone reflected in the pressure– diameter curves (Figure 7B).

In the presence of the Rho‐kinase inhibitor fasudil, myogenic tone was low in cerebral and tongue arteries (Table 2); in arteries from skeletal muscle, tone was fully inhibited by fasudil. All arteries increased tone in response to noradrenaline and serotonin (Table 2).

**TABLE 2:** Tone in isolated small arteries from three giraffe organs.

	Effect of 10 μM noradrenaline and 10 μM serotonin on tone development in addition to myogenic tone (%)		Myogenic tone (%) at different transmural pressures in the presence of the Rho kinase inhibitor Fasudil		
	Noradrenaline	Serotonin	80 mmHg	160 mmHg	260 mmHg
Cerebral (*n* = 5)	22 ± 7	33 ± 12	13 ± 5	9 ± 4	6 ± 2
Tongue (*n* = 6)	49 ± 6	29 ± 6	18 ± 6	18 ± 6	12 ± 4
Skeletal muscle (*n* = 8)	49 ± 7	18 ± 5	6 ± 3	1 ± 2	−1 ± 2

*Note:* Results are agonist‐driven or myogenic tone‐related reductions in diameter (*D*_pas_ − *D*_act_) in percent of passive diameter (*D*_pas_), that is, ((*D*_pas_ − *D*_act_)/*D*_pas_) × 100%. See text for details.

### 3.7 Sympathetic Innervation of Brain Arteries

In small intra‐parenchymal arteries from five giraffe brains, staining for tyrosine hydroxylase immunoactivity revealed its presence in the adventitial layer with a weaker positive reaction in the medial layer (example Figure S8). Therefore, sympathetic innervation of these structures is likely.

## 4 Discussion

New insights from this study include that lowering of the head for drinking was associated with decreases in proximal carotid blood pressure and heart rate, and that drinking per se led to oscillatory increases in proximal jugular pressure, likely driven by the esophageal movements. Concomitantly, cephalic vascular resistance increased, probably via myogenic as well as neuronal mechanisms reducing the intracranial transmural capillary pressure gradients, which were further reduced by increased cerebrospinal fluid pressure. The measured rise in cerebrospinal fluid pressure, however, did not follow the predicted rise due to gravity. The effect of the increased cerebrospinal fluid pressure is analogous to the effect of the high renal interstitial pressure on renal filtration pressure [11]. Surprisingly, during drinking, the initial decrease in proximal carotid pressure reversed in most animals.

Overall, cardiovascular adaptations during drinking in the giraffe are almost exclusively accomplished through exaggerated versions of the well‐known and ubiquitous cardiovascular regulators in mammals.

### 4.1 Hemodynamics of Drinking

Lowering of the head led to decreases in heart rate. Lowering of the head to hoof level was followed by a marked decrease in mean proximal neck arterial pressure together with an increase in distal neck arterial pressures; the latter increased by 100/77 mmHg (systolic/diastolic), corresponding to blood columns of 1.2 and 1.0 m, respectively. As our giraffes on average were about 3.6 m tall, the movement of the head relative to the heart was at least 2 m. Therefore, the pressure increase is considerably smaller than what would be predicted by gravity alone. The bradycardia may be triggered by the carotid baroreceptors localized at the terminal carotid segment near the origin of the vestigial internal carotid and the occipital artery [12], and/or due to a decrease in cardiac preload by sequestration of a sizeable part of the blood volume in the long jugular veins [7]. The present results do not allow distinction between these possibilities, and there are no available data on hemodynamic (Frank–Starling law) versus neurohumoral control of cardiac performance in giraffes. Any explanation should accommodate that drinking per se was followed by increases in both proximal jugular venous pressure and proximal neck arterial pressure, concomitant with a sustained reduction in heart rate. The dermis of the giraffe neck is thick (5–10 mm) and rich in collagen fibers [13], the jugular veins possess valves [7], and we found that distal jugular venous pressure was markedly elevated during drinking, but not sufficient to drive flow towards the heart against gravity. It seems most likely, therefore, that esophageal peristalsis drives jugular venous blood towards the heart during drinking at flow rates sufficient to affect cardiac performance. We are not aware that this mechanism has been described for any other animal. The finding that the frequencies of pressure oscillations in the jugular vein did not match heart rate and the movement of the neck skin during drinking (Video S1) seems consistent with this notion. Further support for an important impact of cardiac preload is provided by our finding that a minor reduction in circulating blood volume (10%) substantially reduced proximal carotid pressure (see below). The most likely explanation for the increase of proximal carotid pressure during drinking seems to be that, under the conditions of dilated neck veins possessing valves, an increased tissue pressure, and a neck dermis with low compliance, the esophageal peristalsis unavoidably drives centripetal blood flow in the jugular veins.

Admittedly, the proximal carotid probe was not necessarily placed in close proximity to the aortic arch. Nonideal probe positioning, therefore, could offset a decrease in proximal carotid pressure. However, it seems unlikely that this deviation is able to undermine our interpretation.

Remarkably, in one giraffe, we serendipitously measured proximal carotid pressure while the animal lowered the head to drink from an empty water reservoir. In this case, the decline in proximal carotid pressure persisted throughout the period of apparent intention to drink, that is the head was at hoof level, supporting a causal relation between the drinking process per se and the reversal of the initial decrease in proximal carotid pressure during drinking.

### 4.2 Resistance Vessels: Active Constriction

Our data on the vascular resistance of the cephalic circulation during drinking come from the carotid and jugular pressures and carotid flows measured during many drinking episodes, but in only two giraffes. In both animals, the index of resistance increased markedly when the head was lowered and decreased transiently when the head was lifted after drinking. Although these results come from measurements obtained only in quasi‐steady states, they underscore the importance of resistance arteries in the protection against high cephalic capillary pressure during drinking, as well as against hypoperfusion immediately after drinking. Our finding of elevated resistance when carotid pressure increases complements the data on flow autoregulation in the giraffe neck reported by McCalden et al. [14].

The cephalic vasoconstriction during lowering of the head may be caused by sympathetic nerve activity and/or myogenic constriction. In vitro, the cerebral, tongue, and muscle small arteries constricted in response to the neurotransmitters noradrenaline and serotonin, and the Rho‐kinase inhibitor fasudil inhibited the myogenic tone. It is likely, therefore, that sympathetic innervation of the vessels modulates cephalic blood flow and resistance, and that, when exposed to the high pressure, baroreceptors mediate sympathetic outflow to the arteries in the head and upper neck leading to vasoconstriction. In support, we found tyrosine hydroxylase to be present, and therefore—most likely—sympathetic nerves to operate in the walls of the small cerebral arteries.

The isolated small arteries from the three cephalic vascular beds responded to increases in transmural pressure with increased tone, that is, myogenic constriction. In vivo, such constriction protects capillaries against increases in arterial pressure and constitutes an important element in the autoregulation of blood flow. Small arteries from the tongue and muscle showed the strongest myogenic constriction around transmural pressures of 200–250 mmHg. Thus, in these vessels, the in vitro maximal response occurred at transmural pressure gradients relevant to drinking. Small cerebral arteries showed the strongest myogenic response at transmural pressures of some 100 mmHg. This may be equally relevant to function in vivo because the lowering of the head is associated with marked increases in the pressure of the cerebrospinal fluid, reducing the cerebral capillary transmural pressure. Different cephalic tissues, therefore, provided small arteries in which the myogenic response characteristics differed, seemingly reflecting the different working conditions in vivo.

The decrease in hydrostatic pressure associated with elevation of the head from a horizontal to an upright position—reported here—was less than expected due to gravity. Similar observations have been made in humans and may reflect collapse of the jugular vein [15, 16], which does occur in giraffes when the head is lifted [7]. In addition, it is possible that during head lifting the decrease in capillary pressure may be important for the fall in cerebrospinal fluid pressure; see review by Oreskovic et al. [17].

### 4.3 Blood Volume and Hemodynamic Resilience

The giraffe blood volume (BlV) of 56 mL/kg body mass (BM) is lower than that of other mammals. In (sedentary) horses of similar BM, BlV measured by Evans's blue is 90 mL/kg BM [18]. In cattle, values of 60–70 mL/kg BM have been reported [19, 20, 21]. Allometric analysis across laboratory animals and man indicates that BlV relates to BM by BlV = 71.5 x BM^1.01^ [22] providing a value of 76 mL/kg BM for 500 kg BM. Thus, in giraffes, blood volume is ≈15%–25% smaller than expected.

The 30% decrease in arterial pressure during controlled hemorrhage of 10% of blood volume seems extreme. In laboratory animals and man, arterial pressure does not change during acute blood losses of less than 15% of blood volume (see reviews [23, 24]). Data from other species are less convincing, but our results in giraffes demonstrate unexpected inadequacy of major compensatory responses to bleeding. The functional blood loss associated with the head‐down position is sizeable. In anesthetized giraffes, jugular venous flow ceased transiently when the head was lowered to heart level [7]; integration of flow over time indicated a sequestration in the jugular veins of some 1.2 L of blood in the head‐down position [7]. In our slightly larger animals, this corresponds to about 1.4 L or ≈5% of measured blood volume. The high sensitivity of blood pressure to modest changes in blood volume indicates unusual limitations of the primary compensatory response, that is, increases in peripheral resistance and cardiac output, the latter being determined by heart rate and preload. Clearly, other parts of our study have shown that heart rates can increase markedly. However, low values of venous compliance—as found in large parts of the jugular veins—limit the option of maintenance of cardiac preload by reduction in venous volume. The high compliance seen in the distal segment at low pressures only is consistent with findings in veins from other mammals. The consequence of this only being the case in the distal segment is unclear. However, high compliance in only the distal part of the jugular vein might allow sequestration during head‐down positions of fractions of the blood volume suitable for reduction of cardiac preload and arterial pressure (see video 2 in Brondum et al. [7]). Although jugular compliance is important for the redistribution of blood during drinking, evidently other factors, for example, venous tone and the rather rigid neck skin, contribute to the function of the jugular veins during drinking.

The large hemodynamic excursions when awakening from anesthesia, where heart rate increases from about 30 to almost 200 beats/min and transient systolic pressures exceed 400 mmHg, are remarkable, but not unique to the giraffe; human weightlifters generate arterial pressures up to 400 mmHg during leg press [25]. It is likely that across species, the cardiovascular system is able to withstand short episodes of intravascular pressures vastly exceeding those occurring normally, and the hemodynamic excursions associated with drinking are much less than those seen during the immediate recovery from anesthesia.

## 5 Conclusion

In summary, giraffes counteract the orthostatic challenges of moving their heads down and up by using well‐described physiological mechanisms: baroreceptor reflexes, precapillary vasoconstriction, and vasodilation. However, when they lower their heads, significant amounts of blood are stored in the neck veins. This reduces venous return and consequently blood pressure and thus counteracts the increase in cephalic arterial pressure by a mechanism unique to this species. It seems surprising that the act of swallowing increases venous return and consequently increases blood pressure. However, the well‐developed, differentially adapted autoregulation of the head vasculature appears sufficient to withstand the high arterial pressure during drinking.

## Author Contributions

**Christian Aalkjær:** conceptualization, investigation, funding acquisition, writing – original draft, methodology, validation, writing – review and editing, formal analysis, project administration, supervision. **Mads Damkjær:** investigation, writing – original draft, methodology, validation, visualization, writing – review and editing. **Ulrik T. Baandrup:** conceptualization, investigation, methodology, writing – review and editing. **Mads F. Bertelsen:** conceptualization, investigation, methodology, writing – review and editing. **Torbjørn Brøgger:** investigation, methodology, validation. **Emil Brøndum:** conceptualization, investigation, methodology, validation. **Carl C. Danielsen:** conceptualization, investigation, validation, methodology, visualization. **Jonas A. Funder:** conceptualization, investigation, methodology, validation. **Carsten Grøndahl:** conceptualization, investigation, funding acquisition, methodology, validation, visualization. **J. Michael Hasenkam:** conceptualization, investigation, funding acquisition. **Per G. Henriksen:** conceptualization, investigation, methodology, validation. **Niels H. Secher:** conceptualization, investigation, writing – original draft, methodology, validation. **Nini Skovgaard:** investigation, methodology. **Morten H. Smerup:** conceptualization, investigation, funding acquisition. **Niklas Telinius:** investigation, methodology, validation. **Kristine H. Østergaard:** conceptualization, methodology, validation. **Peter Bie:** conceptualization, writing – review and editing, methodology, validation. **Tobias Wang:** conceptualization, investigation, funding acquisition, writing – original draft, methodology, validation, visualization.

## Conflicts of Interest

The authors declare no conflicts of interest.

## Supporting information


**Figure S1.** Example of distal and proximal carotid pressures and head position in a giraffe. At the lowest values the neck is horizontal as indicated with the upper hatched line and picture; at the highest values the neck is in an upright position, as indicated by the lower hatched line and picture. The tracing is typical for recordings obtained in four giraffes.

**Figure S2.** Proximal carotid pressure in 7 giraffes before they bend the neck to drink (‘Before drinking’), with the head at ground level ready to drink (‘Start drinking’) and immediately before lift of the head after drinking (‘End drinking’). Mean values and SEM in red analyzed by ANOVA followed by Tukey's test; **p < 0.01, ^o^ *p* > 0.05.

**Figure S3.** Proximal carotid pressure in the giraffe shown in Figure 3 when lowering the head with apparent intention to drink from an empty pond. Head position: black line, right axis; at the lowest position, the head is near the ground as indicated by the lower hatched line and picture; at the highest position, the neck is in an upright position indicated by the upper hatched line and picture.

**Figure S4.** Proximal and distal carotid (red) and jugular (blue) pressures in a drinking giraffe. Head position (black lines) shown on right *y*‐axis; at the lowest values the head is near the ground as indicated with the upper hatched line and picture; at the highest values the neck is in an upright position, as indicated with the lower hatched line and picture. Tracings are unedited; sudden, large excursions shortly after middle of drinking period represent artifacts.

**Figure S5.** Frequency of simultaneous pressure oscillations in carotid artery and jugular vein during drinking. Individual data pairs from 24 observations in six giraffes. Paired *t*‐test; ***p < 0.005.

**Figure S6.** Cerebrospinal fluid pressure in cisterna magna in three anesthetized giraffes maintained in a prone position while the head was moved to vary its position above the heart. The slope of the blue hatched line indicates the hydrostatic effect of gravity in an open system. Mean values and SEM are indicated in red.

**Figure S7.** (A) Mean load‐strain relationships for proximal (red), middle (blue) and distal (black) segments of jugular vein rings from seven giraffes. Vertical lines indicate SEM. Crosses indicate the parameters for maximal load (i.e., where the rings ruptured; maximal strain ± SEM, maximal load ± SEM). The figure to the right a Y‐expanded version of the lower 4% of the left figure. (B) Volume change (V/V_0_) vs. luminal pressure derived from the load‐strain relationships.

**Figure S8.** Isolated brain parenchymal artery stained for tyrosine hydroxylase. Staining pattern typical of arteries from five giraffes.

**Text S1.** Supporting Information.

**Video S1.** Shows drinking giraffe where peristaltics during drinking can be seen oscillating down the lateral neck.

## Data Availability

All data underlying the study are available in the manuscript and the Supporting Information, and there are no restrictions on availability. Aside from the Supporting Information, no additional data files are associated with the manuscript.
