# Supplementary figures and images for: Hemodynamics and Drinking in the Giraffe
Source: Acta Physiol (Oxf). 2025 Apr 22;241(5):e70046. doi: 10.1111/apha.70046 (PMC12012874; doi:10.1111/apha.70046)

Fig. S1

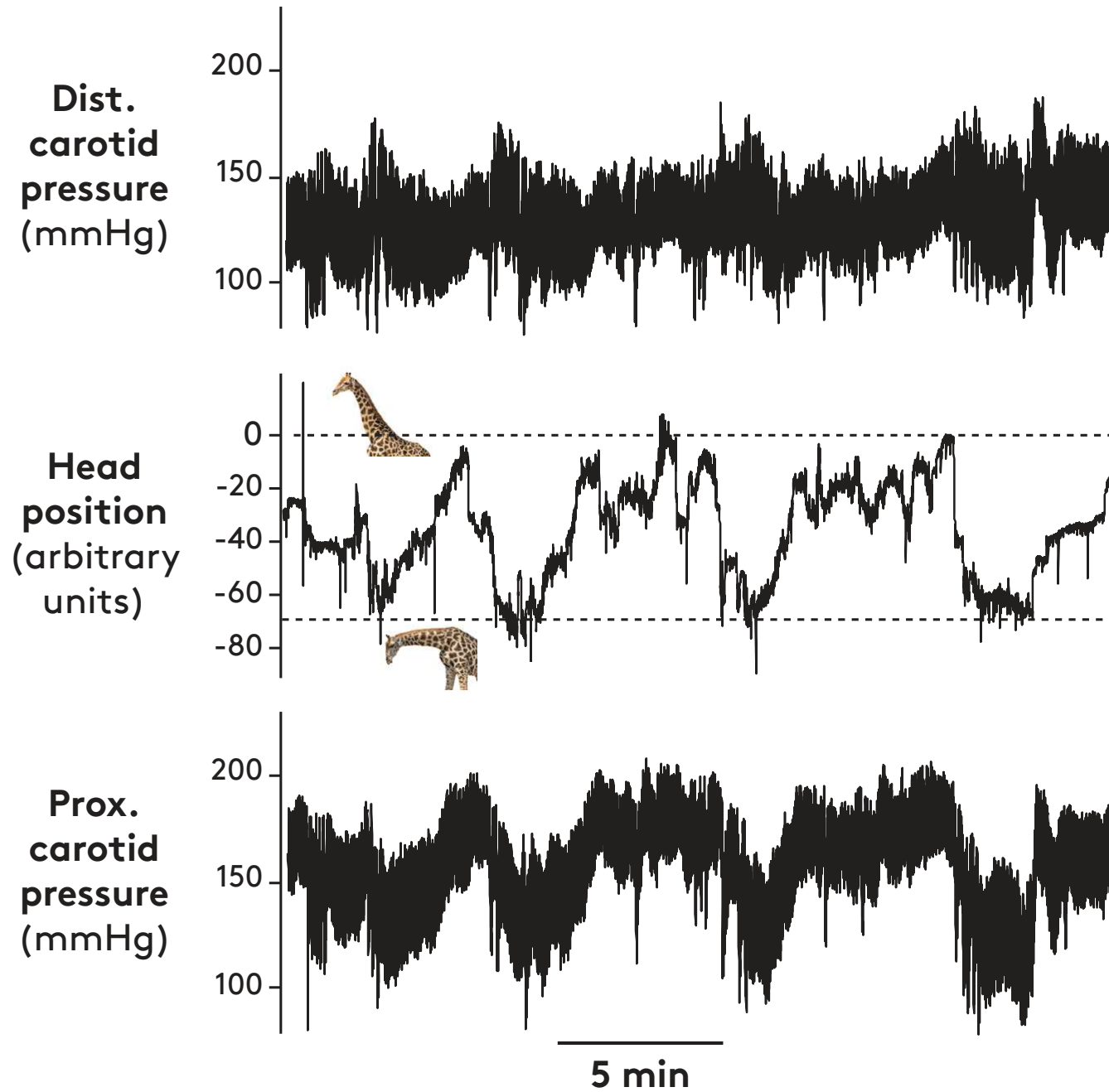

Supplement: Supplementary file 1 — Figure S1. Example of distal and proximal carotid pressures and head position in a giraffe. At the lowest values the neck is horizontal as indicated with the upper hatched line and picture; at the highest values the neck is in an upright position, as indicated by the lower hatched line and picture. The tracing is typical for recordings obtained in four giraffes. [file APHA-241-e70046-s005.pdf]

Fig. S2

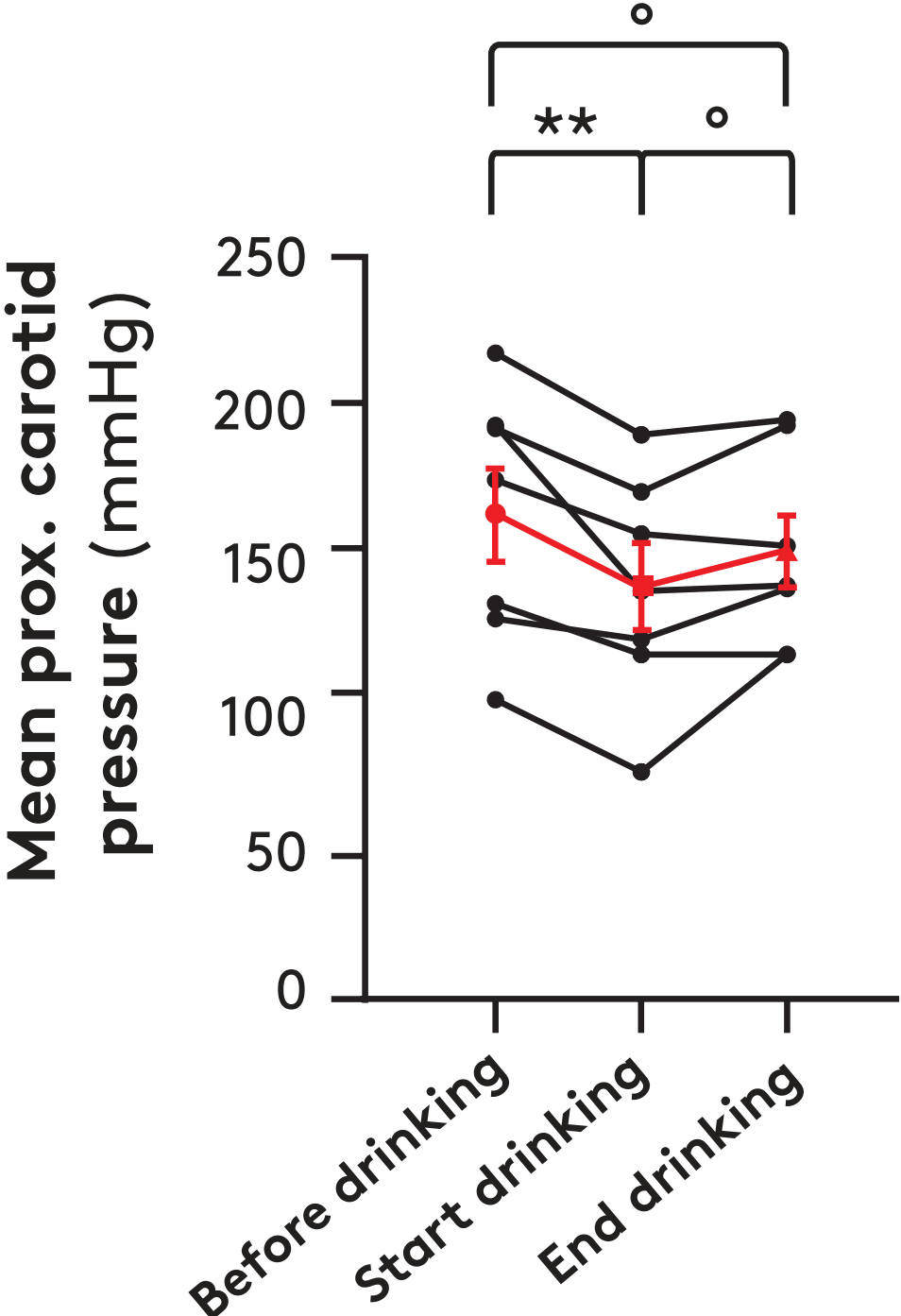

Supplement: Supplementary file 2 — Figure S2. Proximal carotid pressure in 7 giraffes before they bend the neck to drink (‘Before drinking’), with the head at ground level ready to drink (‘Start drinking’) and immediately before lift of the head after drinking (‘End drinking’). Mean values and SEM in red analyzed by ANOVA followed by Tukey's test; **p < 0.01, o p > 0.05. [file APHA-241-e70046-s010.pdf]

Fig. S3

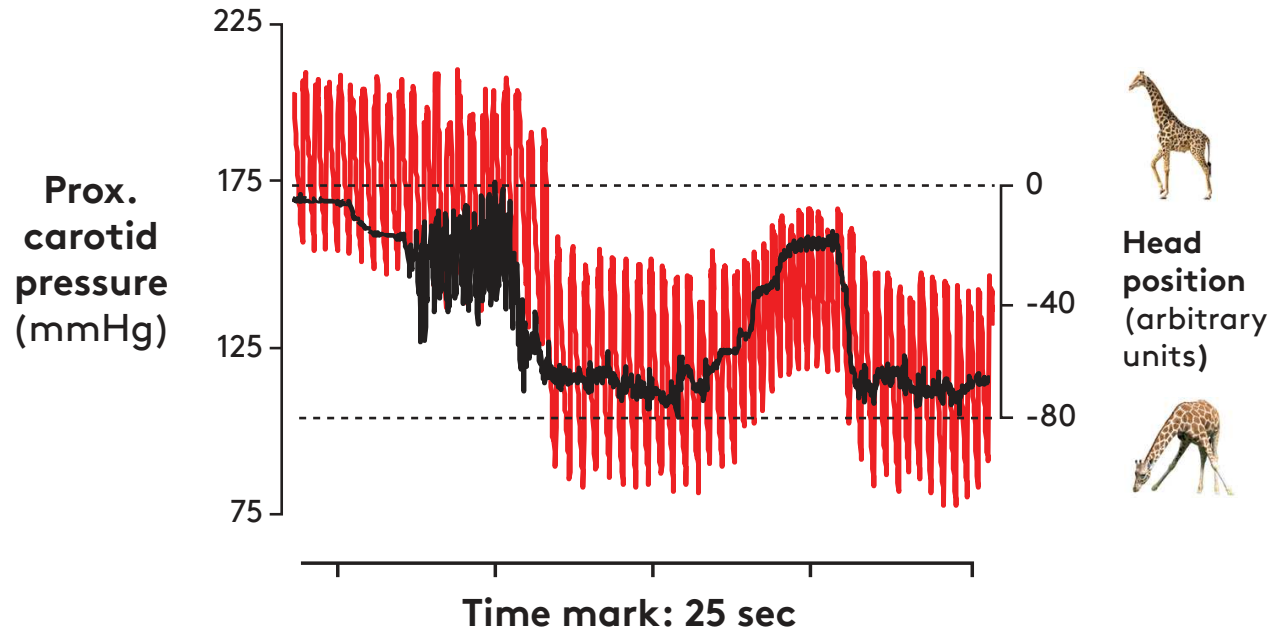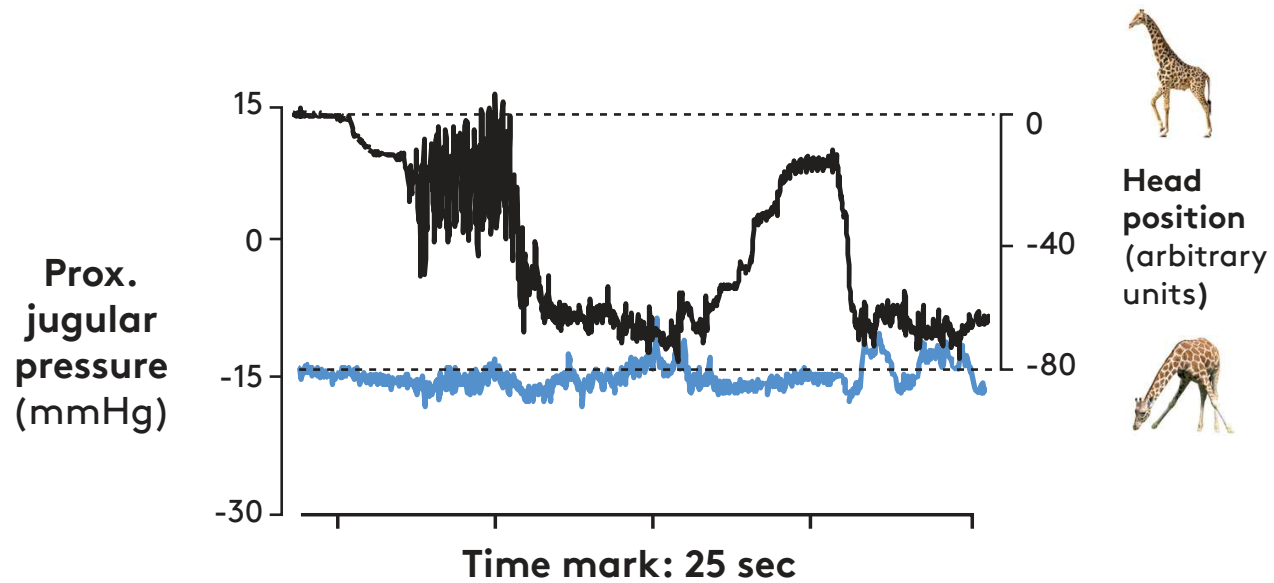

Supplement: Supplementary file 3 — Figure S3. Proximal carotid pressure in the giraffe shown in Figure 3 when lowering the head with apparent intention to drink from an empty pond. Head position: black line, right axis; at the lowest position, the head is near the ground as indicated by the lower hatched line and picture; at the highest position, the neck is in an upright position indicated by the upper hatched line and picture. [file APHA-241-e70046-s001.pdf]

Fig. S4

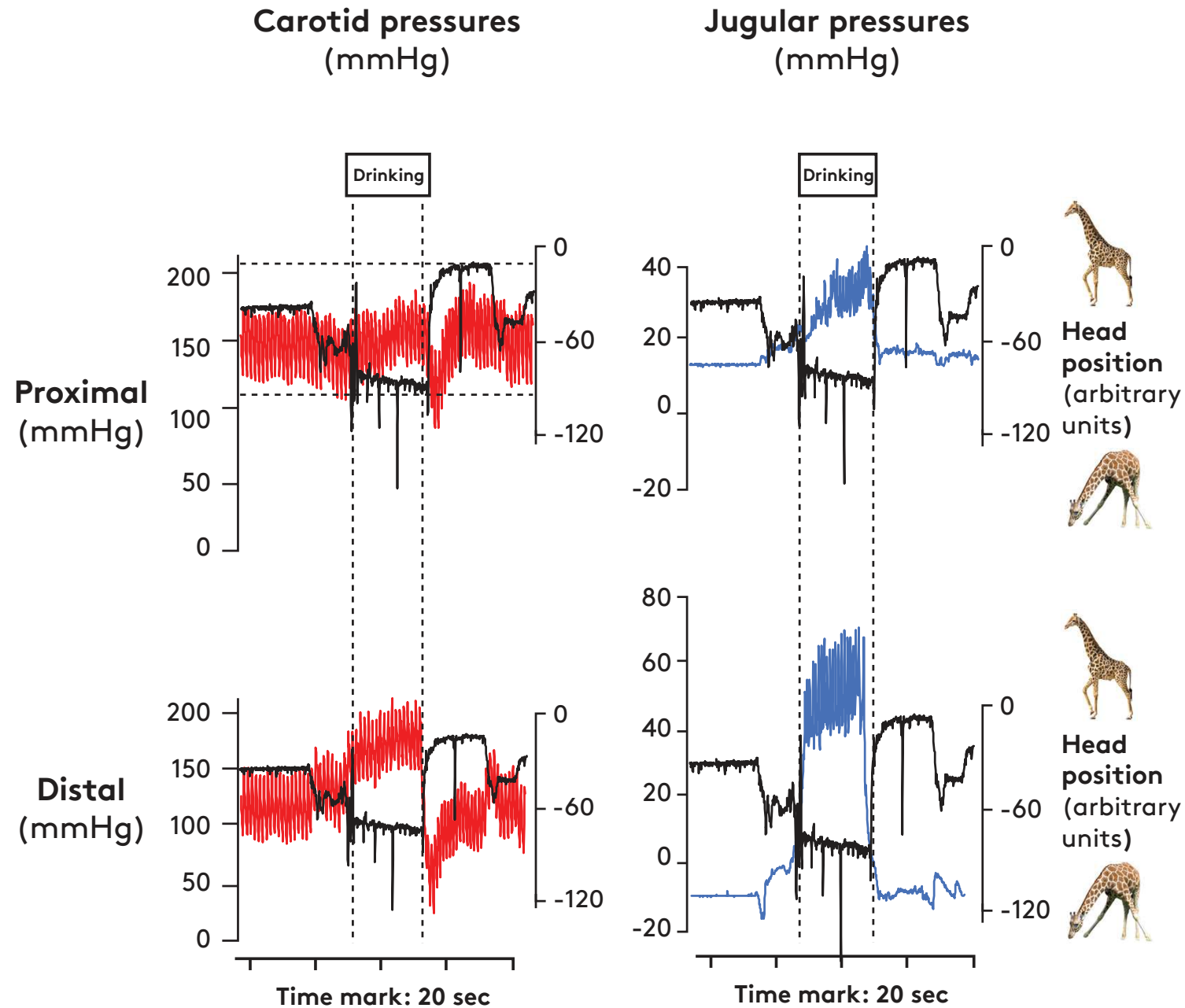

Supplement: Supplementary file 4 — Figure S4. Proximal and distal carotid (red) and jugular (blue) pressures in a drinking giraffe. Head position (black lines) shown on right y‐axis; at the lowest values the head is near the ground as indicated with the upper hatched line and picture; at the highest values the neck is in an upright position, as indicated with the lower hatched line and picture. Tracings are unedited; sudden, large excursions shortly after middle of drinking period represent artifacts. [file APHA-241-e70046-s009.pdf]

Fig. S5

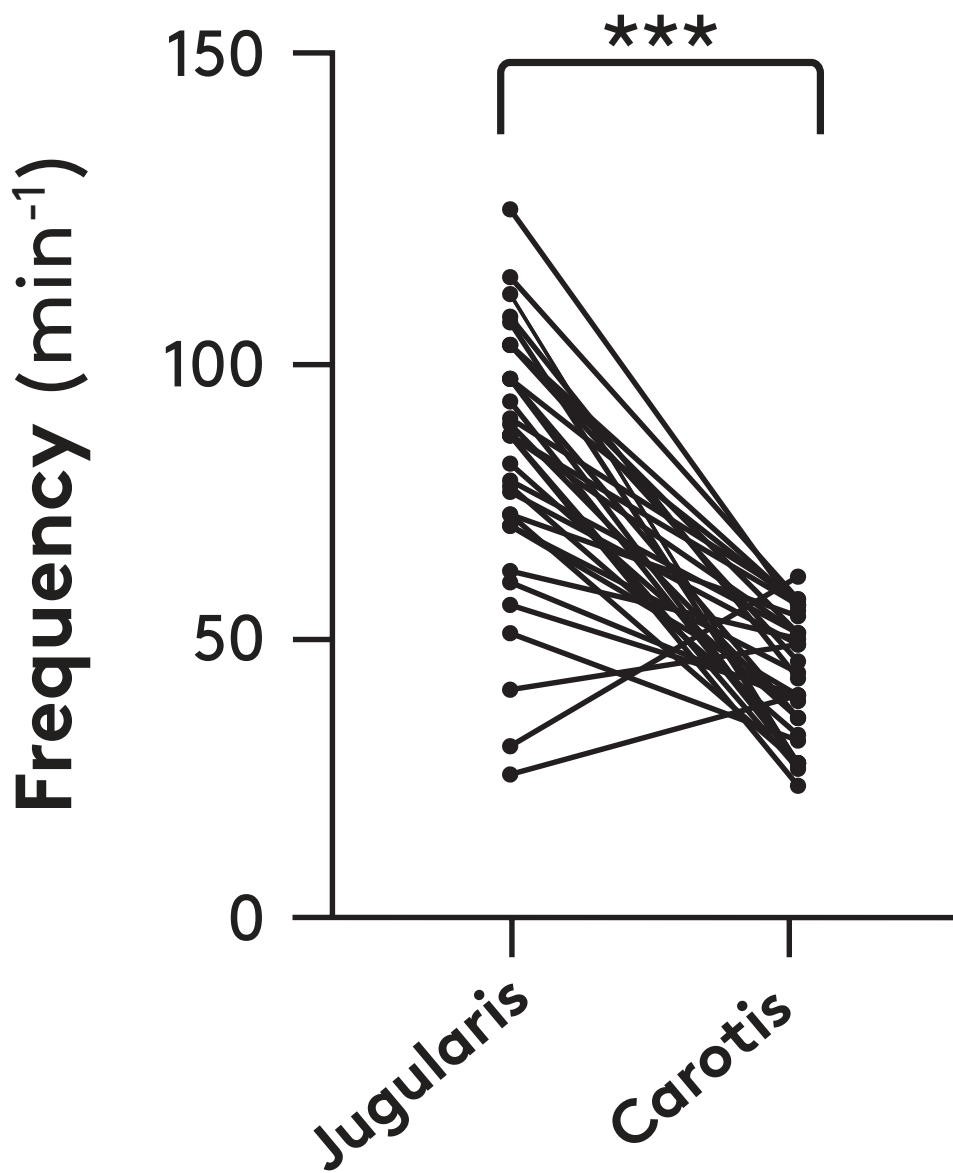

Supplement: Supplementary file 5 — Figure S5. Frequency of simultaneous pressure oscillations in carotid artery and jugular vein during drinking. Individual data pairs from 24 observations in six giraffes. Paired t‐test; ***p < 0.005. [file APHA-241-e70046-s006.pdf]

Fig. S6

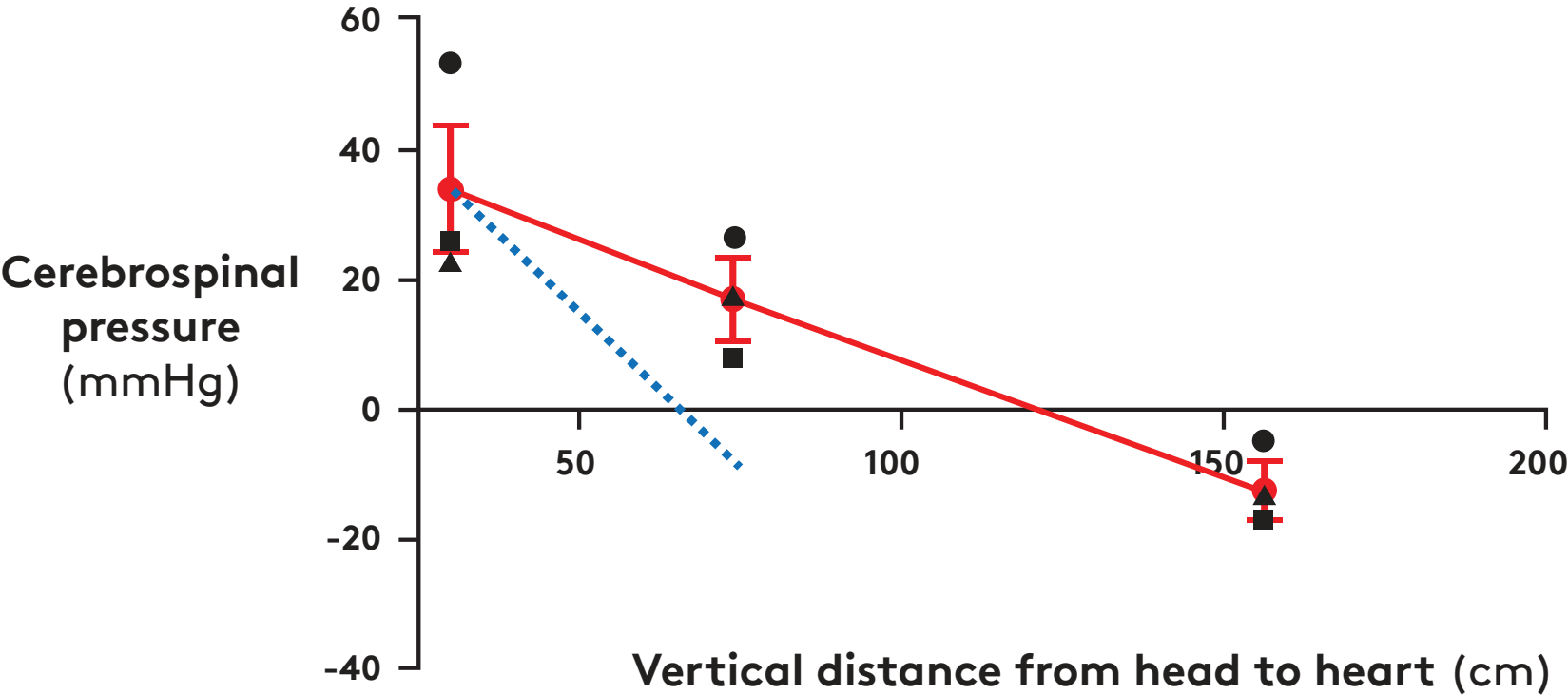

Supplement: Supplementary file 6 — Figure S6. Cerebrospinal fluid pressure in cisterna magna in three anesthetized giraffes maintained in a prone position while the head was moved to vary its position above the heart. The slope of the blue hatched line indicates the hydrostatic effect of gravity in an open system. Mean values and SEM are indicated in red. [file APHA-241-e70046-s008.pdf]

Fig. S7

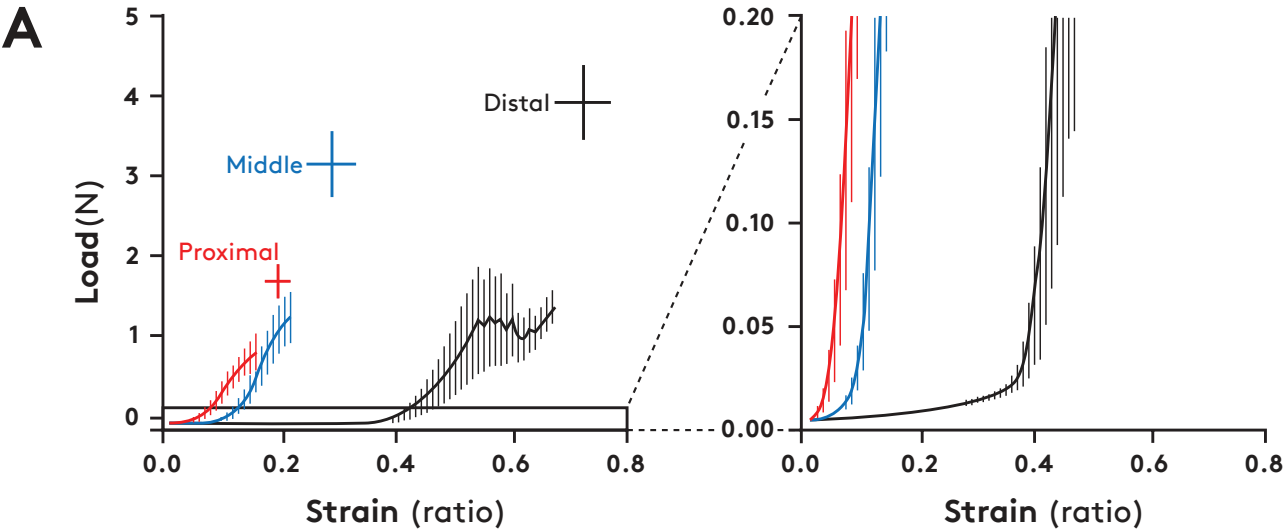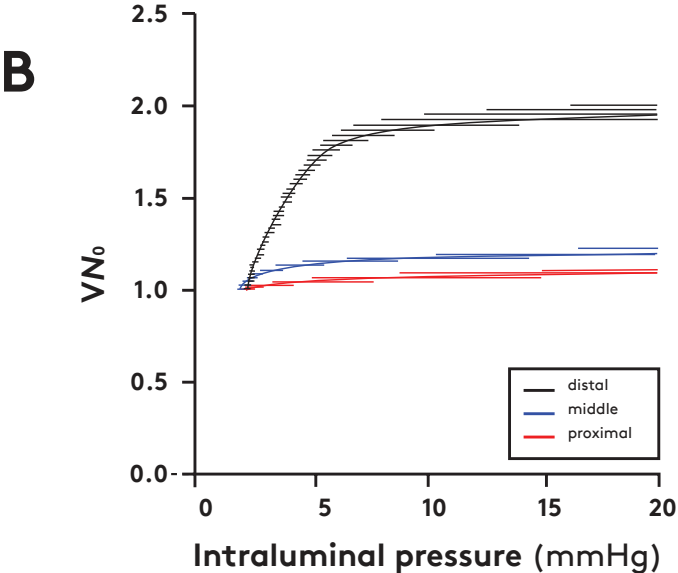

Supplement: Supplementary file 7 — Figure S7. (A) Mean load‐strain relationships for proximal (red), middle (blue) and distal (black) segments of jugular vein rings from seven giraffes. Vertical lines indicate SEM. Crosses indicate the parameters for maximal load (i.e., where the rings ruptured; maximal strain ± SEM, maximal load ± SEM). The figure to the right a Y‐expanded version of the lower 4% of the left figure. (B) Volume change (V/V0) vs. luminal pressure derived from the load‐strain relationships. [file APHA-241-e70046-s002.pdf]

Fig. S8

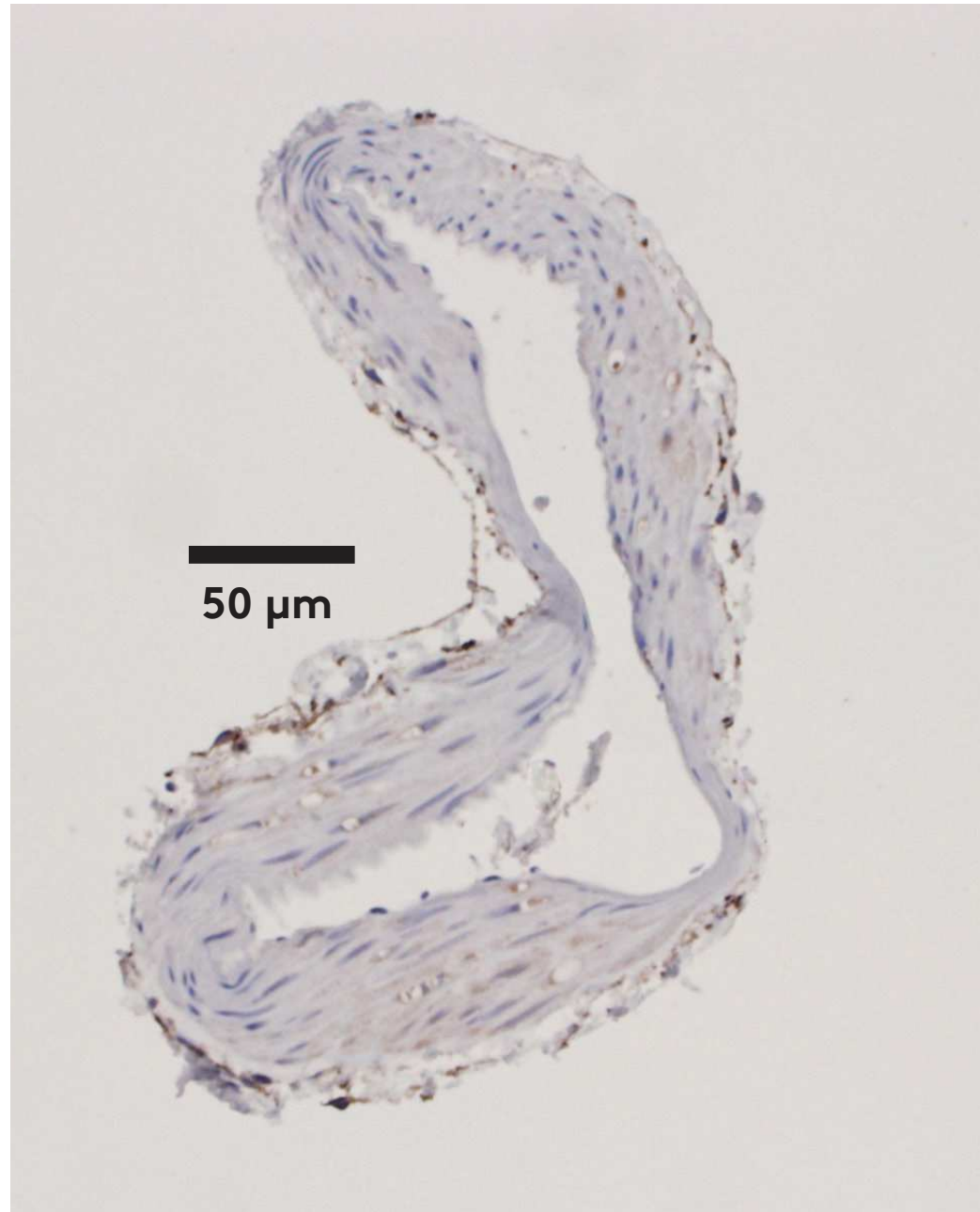

Supplement: Supplementary file 8 — Figure S8. Isolated brain parenchymal artery stained for tyrosine hydroxylase. Staining pattern typical of arteries from five giraffes. [file APHA-241-e70046-s004.pdf]
