# Supplementary material for: Hemodynamics and Drinking in the Giraffe
Source: Acta Physiol (Oxf). 2025 Apr 22;241(5):e70046. doi: 10.1111/apha.70046 (PMC12012874; doi:10.1111/apha.70046)
Supplement: Supplementary file 9 — Text S1. Supporting Information. [file APHA-241-e70046-s007.docx]

**Supporting Information: Text S1**

**Methods S1**

**Plasma volume determination**

Plasma volume was determined by dilution of Evans Blue (EB). Two catheters were placed in the left jugular vein: one proximally for bolus injection of EB and one distally for blood sampling. First, a 50 ml blood sample was obtained for determinations of EB ‘plasma blanks’, hematocrit, plasma osmolality, and electrolyte concentrations. Next, a bolus of EB (Sigma-Aldrich Denmark, Søborg, Denmark; 2 mg/ml, ≈0.5 mg/kg in isotonic glucose) was injected, and the catheter flushed. The exact dose was determined by weighing the syringes. At least six blood samples were obtained over at least 60 min. Blood was centrifuged immediately and plasma EB concentrations determined within hours. For each giraffe, absorbances of initial plasma samples at 627 and 740 nm provided individual plasma blank values ^10^; animal-specific standard curves were obtained by adding known quantities of EB. Plasma volume was calculated from the dose of EB and its initial plasma concentration determined by the log-linear intercept as described ^10^. Blood volume was calculated as plasma volume divided by (1 – hematocrit) ^11^. Hematocrit was determined by centrifugation at 10,000 x g for 15 min and corrected for 2% trapped plasma ^10^.

**Vascular compliances**

The load-strain relationship of carotid artery and jugular veins was determined in specimens obtained from seven giraffes as described ^12^ allowing calculation of vascular compliance. At autopsy, an average of three 2 mm circular rings of giraffe jugular veins were prepared from each of three neck locations: proximal, middle, and distal. The specimens were mounted in a custom-built testing machine on two parallel hooks, the distance between which was increased by 1 mm/s until rupture. Load (F in Newton (N)) and deformation were recorded. The original luminal circumference (l_0_) was defined as the circumference at a small load (0.004 N). Strain (ε) is incremental luminal circumference divided by original circumference, (l - l_0_)/l_0_. The compliance (relative volume change per unit change in applied transmural pressure) was calculated from volume change, V/V_0_ = (1+ε)^2^, and pressure difference, ΔP = (2πF/(l_0_(1+ε)h)/133.3, where h is ring height (2 mm).The derivation of the formulae and the methods of determination of elastin and collagen contents have been described ^12^.

**Function of small cephalic arteries**

Following euthanasia, biopsies from brain parenchyma near the middle cerebral artery, the tongue, and a muscle in the upper neck were obtained, and small arteries dissected under a stereomicroscope. Vessel segments without branches, about 3 mm long and some 300-500 µm in diameter, were mounted in a myograph (DMT, Aarhus, Denmark) for isobaric recording of diameter at 37°C ^13^. The arteries were cannulated at both ends on glass micropipettes filled with a physiological salt solution (PSS; in mM: NaCl 119, KCl 4.7, KH_2_PO_4_ 1.18, MgSO_4_ 1.17, NaHCO_3_ 25, CaCl_2_ 1.6, EDTA 0.026, glucose 5.5; in ‘calcium-free PSS’ the CaCl_2_ was omitted). Solutions were gassed with 5% CO_2_ in air and adjusted to pH 7.4. Occlusion of the distal pipette permitted experiments under no-flow conditions. The proximal pipette was connected to a pressure regulator (Mini Pressure Regulator, DMT, Aarhus, Denmark) to control transmural pressure. The myograph was placed on an inverted microscope (Leica, Wetzlar, Germany) equipped with a CCD camera (SONY, XC-75CE, Tokyo, Japan), and the external diameter of the vessels measured from video images of the transilluminated preparation.

The cannulated arteries were exposed to 80 mmHg transmural pressure for ≈30 min. Then, a pressure-diameter curve was obtained by first reducing transmural pressure to 20 mmHg and subsequently increasing it in 20 mmHg steps to 220 mmHg (for cerebral arteries) or 260 mmHg (for other arteries). Next, the transmural pressure was returned to 80 mmHg and the response to 10 µM noradrenaline and 10 µM serotonin obtained. Then, a new pressure-diameter curve was established at pressures of 80, 160, and 260 mmHg and the pressure-diameter relationship repeated with 1 µM of the Rho-kinase inhibitor fasudil. Subsequently, under superfusion with Ca^2+^-free PSS with 30 µM papaverine, a passive diameter (D_pas_) pressure curve was established (from 20 to 220 mmHg (cerebral arteries) or 260 mmHg (other arteries)). At each transmural pressure, vessel diameter was recorded at steady state. Vessel tone (myogenic or agonist induced) is expressed as vessel diameter, D_act_, at a given pressure relative to the passive vessel diameter in calcium-free solution with papaverine at the same pressure (D_pas_). Results are active reductions in diameter (D_pas_ – D_act_) in percent of passive diameter (D_pas_), i.e., ((D_pas_ – D_act_)/D_pas_) x 100%.
